# Hemiarthroplasty as a salvage treatment for failed reverse total shoulder arthroplasty

**DOI:** 10.1016/j.jseint.2021.07.003

**Published:** 2021-09-04

**Authors:** Philipp Kriechling, Octavian Andronic, Karl Wieser

**Affiliations:** Department of Orthopedics, Balgrist University Hospital, Zürich, Switzerland

**Keywords:** Reverse total shoulder arthroplasty, Salvage surgery, Complication, Revision, Hemiarthroplasty, Clinical outcome

## Abstract

**Background:**

The implantation rates of reverse total shoulder arthroplasties (RTSAs) are increasing worldwide, resulting in higher absolute numbers of the associated complications and revision surgeries. This requires the discussion of salvage therapies for failed RTSAs without revision to a new RTSA. Revision to hemiarthroplasty may offer a valid fallback option in certain cases. This study aimed to analyze the incidence, indications, and clinical outcomes, especially the reduction in pain levels compared to a matched control group.

**Methods:**

Our prospectively enrolled patient cohort of RTSA implantations at a tertiary referral center between January 2005 and December 2018 was retrospectively queried for revision to a hemiarthroplasty. For clinical outcome evaluation, a minimum follow-up duration of 2 years after revision to hemiarthroplasty was required. Clinical outcome measures were compared to two matching groups, one with RTSA preserving revision and one without any reintervention. The outcome measures were the absolute and relative Constant-Murley score (aCS and rCS), Subjective Shoulder Value (SSV), range of motion, and pain.

**Results:**

A total of 21 out of 1237 RTSAs (1.7%) underwent salvage revision to hemiarthroplasty at a mean time of 20 ± 21 months (range, 1-75 months). Of those, 12 were available for a minimum follow-up of 2 years after revision to a hemiarthroplasty. The main indications were glenoid loosening (8/12), scapular spine fracture (2/12), and instability (2/21). Clinical outcome was analyzed at a mean follow-up period of 46 ± 26 months (24 months to 123 months) after revision to a hemiarthroplasty. The revision significantly reduced CS pain from 6 ± 4 points to 12 ± 3 points (scale 0 to 15 with 15 as optimum, *P* < .01). The aCS, rCS, SSV, and range of motion did not improve. Comparison with the RTSA preserving revision group and the RTSA group without reintervention showed significantly worse outcome scores for aCS (33 ± 10 vs. 55 ± 19 vs. 69 ± 12 points), rCS (41 ± 14% vs. 67 ± 20% vs. 84 ± 13%), SSV (35 ± 19% vs. 64 ± 20% vs. 81 ± 15%), flexion (53 ± 27° vs. 64 ± 20° vs. 128 ± 24°), and abduction (50 ± 23° vs. 109 ± 42° vs. 142 ± 24°). Pain was similar in all groups at the last follow-up visit.

**Conclusion:**

In a few cases, RTSA retention or revision to another RTSA is impossible. For those patients, conversion to hemiarthroplasty is a valid fallback option to reduce the patient's pain levels and provide low-level function.

The implantation rates of reverse total shoulder arthroplasties (RTSAs) continue to grow exponentially worldwide, clearly surpassing the implantation rates of anatomical shoulder arthroplasties.^10^^,^^36^ The reasons for the increasing incidence might include an aging society^24^^,^^28^ and a variety of indications,^26^ including rotator cuff arthropathy,^31^ massive rotator cuff tear with or without arthritis,^11^^,^^37^ osteoarthritis,^29^ rheumatoid arthritis,^8^ and proximal humeral fracture^17^ and sequelae.^33^ Furthermore, RTSA is increasingly used as a versatile revision option for failed RTSAs,^39^ anatomical arthroplasties, or hemiarthroplasties.^3^^,^^39^

As satisfying outcomes are reported according to the different indications from midterm to long-term follow-up,^2^ a high number of complications and reinterventions still have to be respected and addressed.^7^^,^^12^^,^^40^ Zumstein et al^40^ reported in a systematic review an overall complication rate of 24% and an overall reintervention rate of 13%. The incidence seems to depend strongly on the length of the observation period. Parada et al^32^ reported a reintervention rate of 3% at a mean of 22 months, while Boileau et al^4^ reported 10% at 30 months and Bacle et al^2^ reported 12% at 150 months. The overall aim of necessary revision surgery is an arthroplasty preserving approach.^6^^,^^39^ In some cases, no RTSA retaining revision is possible, leaving resection arthroplasty, glenohumeral arthrodesis, or revision to hemiarthroplasty the only remaining options.^25^^,^^30^^,^^38^^,^^39^ Between those, hemiarthroplasty seems to be a promising revision option for failed RTSAs. In such cases, the main goal is to reduce the pain levels and, if possible, maintain some shoulder function. Newer designs with bipolar and/or oversized heads are currently available to fill the dead space after RTSA removal, and modular arthroplasty systems allow straightforward revision with retention of a well-fixed humeral stem.

Only a few reports on the outcome of hemiarthroplasty after RTSA exist.^13^^,^^16^ Glanzmann et al^16^ reported on 16 patients who received a hemiarthroplasty, primarily for glenoid loosening (11/16) and infection (3/16). The reported postoperative Constant-Murley score was 25 ± 12 points postoperatively. Satisfactory results were achieved in nearly half of the patients; however, the pain level did not improve.

As the RTSA implantation rates are increasing, a substantially higher total number of complications will occur in the future. Some of them will be unable to be revised with the retention of the implant. Therefore, this study aimed to describe the incidence at our institution and analyze the clinical outcomes, especially the pain level, of hemiarthroplasties after failed RTSA compared to matched control groups. We hypothesized that RTSA revision to hemiarthroplasty might be a valuable fallback option with sufficient pain reduction.

## Material and methods

### Ethics approval

This study was approved by the cantonal ethics committee of the University of Zürich (ID 2018- 01494) and conducted following the Helsinki Declaration.

### Patient selection

Our prospectively enrolled institutional database was screened for patients who received an RTSA between January 2005 and December 2018 (in our institution and consecutively needed revision surgery for hemiarthroplasty with a minimum follow-up duration of 2 years). The inclusion criteria were revision to a hemiarthroplasty, signed informed consent obtained, patients older than 18 years, complete basic demographic data present, and complete surgical reports available. For the outcome analysis, complete clinical follow-up (Constant-Murley score and Subjective Shoulder Value)^9^^,^^14^ and radiological follow-up were necessary.

Two matching cohorts in a 1:1 ratio were identified, one group including patients with RTSA retaining revision surgery and one RTSA control group without the need for reintervention. The matching criterion for RTSA retaining control group was the indication for revision surgery with a minimum follow-up duration of 2 years after revision surgery. The criteria for the shoulders without revision surgery were age at primary surgery, indication for RTSA, sex, operated side, American Society of Anesthesiologists score (physical status classification system of the American Society of Anesthesiologists), and body mass index.

### Surgical technique

**Figure 1:**
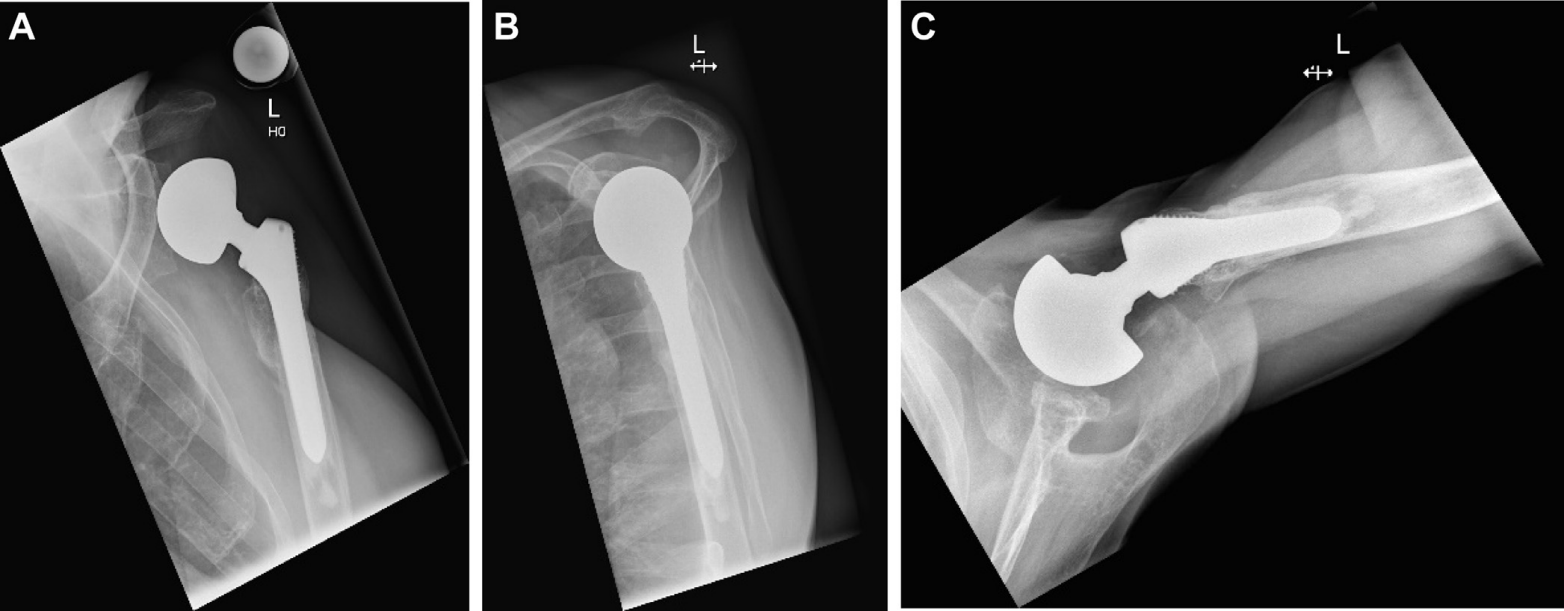
A bipolar big head hemiarthroplasty with stem retention after glenoid loosening in anteroposterior view (**A**), Neer view (**B**), and axillary view (**C**).

Fellowship-trained staff shoulder surgeons performed the total joint replacement in a standardized manner. The implantation technique for primary RTSA was described elsewhere.^22^^,^^23^ For revision surgery, the patient was placed in a beach chair position with general or regional anesthesia using a deltopectoral approach. Antibiotic prophylaxis with cefuroxime 1.5 g (Fresenius Kabi, Switzerland) was administered intravenously 30 minutes before skin incision. In the hemiarthroplasty group, the glenoid component was removed in all patients. Bony glenoid augmentation was used in 7 of 12 patients (5 allograft and 2 tricortical iliac crest bone) using screws in 1 of 7 shoulders and press-fit implantation in 6 of 7 shoulders. The well-fixed humeral stem was left in place in all patients. The primary stem fixation method was cementation in 8 of 12 cases and press-fit in 4 of 12 shoulders. In 7 of 12 cases, a standard hemiarthroplasty head was used; in the other 5 patients, a bipolar big head hemiarthroplasty was implanted (Fig. 1). The subscapularis was refixed in 8 of 12 cases using FiberWire (Arthrex, Naples, FL, USA).

In the RTSA preserving matching group, 2 of 12 patients underwent autologous glenoid augmentation with iliac crest bone and 4 of 12 patients underwent refixation of the subscapularis muscle during revision surgery. The primary stem fixation method was cementation in 7 of 12 cases and press-fit in 5 of 12 cases. The stem was revised in 1 of 12 patients using cementation for a previously uncemented stem. Aftercare consisted of wearing a sling for six weeks with allowance for active range of motion (ROM) exercises in all patients.

### Clinical and radiological follow-up

The patients were followed up at 6 weeks, 18 weeks, and regularly every 1 to 2 years. The follow-up was conducted through an orthopedic staff member specializing in shoulder surgery. All patients underwent clinical examination using the Subjective Shoulder Value^15^ and the absolute and relative Constant-Murley score,^9^^,^^15^ including full assessments of ROM and abduction strength using a validated dynamometer. The radiological examination included standardized X-rays in 3 planes.

### Statistical analysis

Study data were collected and managed using REDCap electronic data capture tools version 8.6 hosted at Balgrist University Hospital.^18^^,^^19^

The statistical analyses were performed using SPSS software v27.0 (IBM, Armonk, NY, USA). The normal distribution of variables was tested with the Shapiro-Wilk test, and preoperative and postoperative scores were compared with the Wilcoxon rank-sum test for nonparametric distribution. Fisher's exact test was used for categorical variables. Analysis of the clinical outcome between the three groups was performed using the Kruskal-Wallis test for nonparametric data with post-hoc Bonferroni correction between the groups. A *P* value of less than 0.05 was considered significant.

## Results

**Figure 2:**
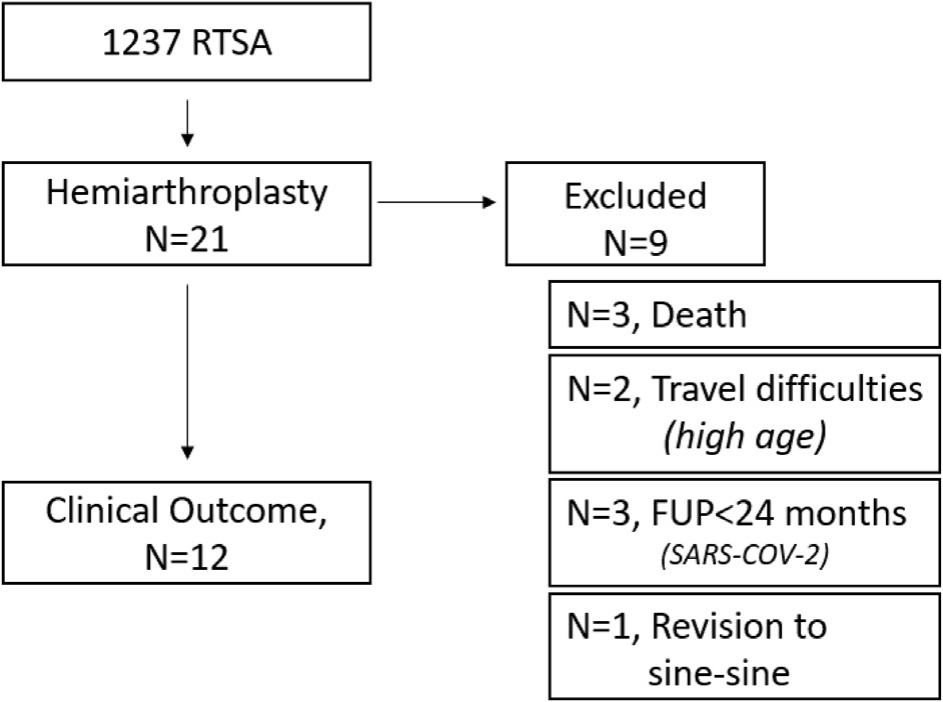
Flowchart of patients included. *RTSA*, reverse total shoulder arthroplasty, *FUP*, follow-up.

Between January 2005 and December 2018, a total of 1237 RTSAs were implanted with reintervention surgery in 161 shoulders. Twenty-one underwent revision surgery for hemiarthroplasty (1.7% of all RTSAs and 13% of all reinterventions) at an average time of 20 ± 21 months (range, 1-75 months) (Fig. 2). A follow-up duration of more than 2 years after revision to hemiarthroplasty was available for 12 patients. Demographic data are summarized in Table I.

**Table I:** Demographic data given as mean ± standard deviation (minimum; maximum) or absolute numbers.

Group	Hemi	Revision RTSA	Control	Hemi vs. Rev RTSA	Hemi vs. control
Number	12	12	12		
Age (yr)	73 ± 7	64 ± 11	72 ± 6	0.03	0.76
Right-sided	6	7	10	0.68	0.19
Female	10 (83%)	6 (50%)	10 (83%)	0.08	1.00
BMI (kg/m^2^)	26 ± 5	29 ± 3	27 ± 4	0.30	0.69
ASA				0.90	0.76
ASA I	0 (5%)	2	0		
ASA II	8 (75%)	6	10		
ASA III	4 (25%)	4	2		

*RTSA*, reverse total shoulder arthroplasty; *Hemi*, hemiarthroplasty; *Rev*, revision; *BMI*, body mass index; *ASA*, American Society of Anesthesiologists score.

### Indications for hemiarthroplasty

Details of the 12 patients with sufficient clinical follow-up are displayed in Table II. All patients with glenoid loosening (8/12) and scapula spine fracture (2/12) refused to undergo revision back to RTSA at a later stage. Two of 12 patients with recurrent instability received the hemiarthroplasty as the final treatment.

**Table II:** Patients available for analysis with a minimum follow-up duration of 24 months after revision to a hemiarthroplasty.

ID	Age	RTSA indication	Hemi indication	Tra	Type	Bone	Conv	FUP
1	75	MRCT	Glenoid loosening with baseplate dislocation from bone	No	Hemi	Allo	14	60
2	75	Failed hemi	Glenoid loosening	No	Hemi	ICBG	75	114
3	84	MRCT	Glenoid loosening	No	Hemi	-	1	124
4	75	Failed TSA	Glenoid loosening	Yes	Hemi	Allo	20	45
5	71	CTA	Scapula spine fracture	Yes	Bipolar	Allo	2	56
6	75	CTA	Glenoid loosening with baseplate dislocation from bone	No	Hemi	-	25	78
7	74	CTA	Glenoid loosening with baseplate dislocation from bone	No	Hemi	Allo	54	78
8	81	CTA	Glenoid loosening	Yes	Bipolar	Allo	14	67
9	72	Failed TSA	Glenoid loosening	No	Hemi	ICBG	23	62
10	74	Failed ORIF	Instability	No	Bipolar	-	2	26
11	64	Osteoarthritis	Scapula spine fracture	No	Bipolar	-	9	59
12	60	MRCT	Instability	Yes	Bipolar	-	6	30

*RTSA*, reverse total shoulder arthroplasty; *Hemi*, hemiarthroplasty; *Tra*, trauma; *Conv*, conversion to hemiarthroplasty; *FUP*, follow-up period; *MRCT*, massive rotator cuff tear; *Allo*, allograft; *ICBG*, iliac crest bone graft; *TSA*, total shoulder arthroplasty; *CTA*, cuff tear arthropathy; *ORIF*, fracture treatment with open reposition and internal fixation using a plate or nail.

Conv displays the duration from implantation of RTSA to conversion to hemiarthroplasty in months. FUP displays the maximum follow-up after RTSA implantation. Bone displays the bone graft used for glenoid augmentation.

### Clinical and radiographic outcome

Detailed clinical results are shown in Table III and Supplemental Table 1. There was no significant change after revision to hemiarthroplasty for aCS, rCS, SSV, and ROM. Comparing those parameters to patients with RTSA retaining revision and patients without the need for reintervention revealed significantly worse results for most of the parameters except for pain, which was comparably low for all three groups at the latest follow-up visit.

**Table III:** Preoperative and postoperative outcome measures as mean ± standard deviation (range min to max) for the RTSA with conversion to hemiarthroplasty, revision RTSA with retaining components, and the control group.

	Hemi	Rev CTL	RTSA CTL	*P* value
Number	12	12	12	
aCS				
Preop RTSA	30 ± 11 (2; 43)	32 ± 9 (15; 43)	35 ± 15 (15; 61)	.82
Preop revision	30 ± 18 (8; 68)	26 ± 13 (15; 48)		.80
Latest FUP	33 ± 10 (15; 55)∗†	55 ± 19 (29; 78)	69 ± 12 (36; 83)	<.01
rCS (%)				
Preop RTSA	39 ± 14 (2; 55)	40 ± 12 (17; 56)	45 ± 17 (24; 74)	.85
Preop revision	39 ± 21 (10; 82)	33 ± 16 (17; 61)		.63
Latest FUP	42 ± 14 (19; 67)∗†	67 ± 20 (35; 90)	84 ± 13 (48; 95)	<.01
SSV (%)				
Preop RTSA	28 ± 18 (5; 60)	27 ± 15 (10; 50)	37 ± 17 (10; 60)	.33
Preop revision	37 ± 25 (0; 80)	27 ± 22 (10; 70)		.25
Latest FUP	35 ± 19 (0; 60)∗†	64 ± 20 (30; 95)	81 ± 15 (50; 100)	<.01
CMS pain				
Preop RTSA	6 ± 4 (0; 15)	8 ± 4 (3; 14)	5 ± 4 (0; 10)	.18
Preop revision	7 ± 4 (2; 11)	7 ± 4 (5; 15)		.79
Latest FUP	12 ± 3 (7; 15)	13 ± 3 (6; 15)	13 ± 3 (8; 15)	.60
Flexion (°)				
Preop RTSA	65 ± 27 (0; 100)	69 ± 38 (20; 140)	97 ± 31 (45; 140)	.06
Preop revision	75 ± 36 (30; 160)	54 ± 25 (30; 100)		.22
Latest FUP	55 ± 27 (0; 90)∗†	102 ± 34 (40; 138)	128 ± 24 (70; 160)	<.01
Abduction (°)				
Preop RTSA	63 ± 25 (0; 90)	65 ± 26 (20; 100)	78 ± 25 (20; 120)	.27
Preop revision	70 ± 39 (30; 160)	53 ± 23 (30; 90)		.40
Latest FUP	50 ± 23 (0; 70)∗†	109 ± 42 (30; 165)	142 ± 24 (95; 170)	<.01
ER (°)				
Preop RTSA	23 ± 26 (−20; 65)	27 ± 26 (−20; 60)	28 ± 25 (−10; 60)	.92
Preop revision	9 ± 30 (−50; 60)	21 ± 23 (−10; 60)		.35
Latest FUP	13 ± 23 (−40; 50)	17 ± 17 (−10; 45)	28 ± 27 (−10; 75)	.36
IR				
Preop RTSA	5 ± 3 (0; 10)	4 ± 2 (2; 10)	5 ± 3 (0; 10)	.87
Preop revision	3 ± 3 (0; 8)	3 ± 2 (0; 6)		.61
Latest FUP	5 ± 3 (0; 10)	4 ± 2 (2; 8)∗	7 ± 2 (4; 10)	.05
Force mean (kg)				
Preop RTSA	0 ± 1 (0; 3)	1 ± 2 (0; 5)	1 ± 1 (0; 3)	.35
Preop revision	1 ± 1 (0; 3)	0 ± 1 (0; 2)		.48
Latest FUP	0 ± 0 (0; 0)∗†	2 ± 2 (0; 6)	3 ± 2 (0; 5)	.01
Follow-up (mo)				
Post revision	46 ± 26 (24; 123)	61 ± 29 (24; 120)		
Post RTSA	66 ± 30 (25; 124)	72 ± 25 (32; 121)	80 ± 33 (27; 120)	.55

*RTSA*, reverse total shoulder arthroplasty; *Hemi*, hemiarthroplasty; *Rev*, revision; *CTL*, control; *aCS*, absolute Constant-Murley score; *preop*, preoperative; *postop*, postoperative; *rCS*, relative Constant-Murley score; *FUP*, follow-up period; *SSV*, Subjective Shoulder Value; *CMS*, Constant-Murley Score; *ER*, external rotation; *IR*, internal rotation.

Internal rotation is defined according to Constant-Murley score rating with 10 for best value, pain is defined according to Constant-Murley score rating with 15 for best value.

∗ Highlights significant findings using pots-hoc Bonferroni test for comparison to the RTSA, control (*P* < .05).

† Highlights significant findings using pots-hoc Bonferroni test for comparison to the revision RTSA, group (*P* < .05).

At the latest radiological follow-up, 6 of 12 patients presented with anterosuperior escape of the implant, and another 3 of 12 with an anterior but not superior position of the implant.

### Complications and reinterventions

Two complications were reported in the hemiarthroplasty group. One fracture of the iliac crest occurred because of bone harvesting for glenoid bone grafting while revising hemiarthroplasty in one patient. The fracture was treated surgically using a plate and healed. One patient presented with a traumatic clavicular fracture at 17 months after implantation of the hemiarthroplasty. The fracture healed without performing surgery.

One patient in the RTSA preserving group presented with traumatic scapular spine fracture 10 months after glenoid component revision for glenoid loosening. The fracture was treated conservatively and healed.

In the RTSA matching cohort without reintervention, one patient reported a transient incomplete lesion of the brachial plexus. Another patient suffered from a superficial wound infection, which was treated with antibiotics and healed. No reintervention surgeries were required.

## Discussion

Because the implantation rates of RTSAs are increasing worldwide, orthopedic shoulder surgeons will ultimately face a rising number of revision surgeries in the future.^12^^,^^40^ As RTSA retaining treatment algorithms were reported to yield satisfying results,^1^^,^^4^^,^^5^^,^^20^^,^^39^ RTSA preserving revision surgery might not always be achievable. If this is no longer feasible, resection arthroplasty, arthrodesis, and salvage hemiarthroplasty are among the remaining therapeutic options.^30^^,^^38^

This study investigated the incidence and clinical and radiological outcomes of hemiarthroplasty after failed RTSA compared to a matching cohort with RTSA retaining revision surgery and patients without reintervention. In cases of necessary implant revision, all efforts are invested in retaining or secondarily reimplanting an RTSA. We identified 21 of 1379 cases (1.7%) that were in ultimate need of a fallback revision to shoulder hemiarthroplasty. Analyzing the patients with a minimum follow-up duration of 2 years after revision to hemiarthroplasty, the main indications were glenoid loosening (8/12), scapula spine fractures (2/12), and instability (2/12). The conversion to hemiarthroplasty led to a significant reduction in pain, with half of the patients reported being completely pain-free. However, the aCS, rCS, SSV, and ROM remained unchanged after revision and were significantly inferior compared to patients with RTSA retaining revision surgery and patients without reintervention.

The main indications for revision to hemiarthroplasty in our patient series were glenoid loosening and scapula spine fractures with insufficient glenoid fixation. Glenoid component failure is one of the most common indications for RTSA revision surgery. Analyzing global indications for reintervention in RTSA, glenoid-related complications were reported to account for approximately 15% of all indications.^1^^,^^4^^,^^20^ Different strategies exist when revising such cases, mostly depending on the quality of the remaining glenoid bone stock. If the bone stock is sufficient, a new long peg or even a custom-made glenoid base plate can be implanted. Insufficient glenoid bone stock may be treated with structural bone grafting if the defect provides some degree of containment. Even bone grafting or custom-made implants are no longer feasible in some cases, leaving no option other than fallback revision with conversion to a hemiarthroplasty.^25^^,^^34^

In a multicenter study including 79 patients analyzing the results of glenoid loosening with a minimum follow-up duration of 2 years, 35% could be treated conservatively, 32% with RTSA retaining glenoid component revision, and 33% with revision to hemiarthroplasty. These high conversion rates to hemiarthroplasty are reflected in our results. In total, 57% of patients with glenoid revision were satisfied, in contrast to only 6% of the hemiarthroplasty patients (Only 50% of the hemiarthroplasty patients were available for analysis.). Analyzing the Constant-Murley pain score, revision of the glenoid component yielded the best results compared to conservative treatment and revision to hemiarthroplasty. Comparison of conservative treatment with hemiarthroplasty showed no difference in pain levels between the groups. However, these results might be biased, as pain is one of the leading reasons for revision in these patients. The reported absolute Constant scores were similarly low, with 41 ± 20 points for the conservatively treated patients, 46 ± 17 points after component revision, and 37 ± 19 points in the hemiarthroplasty group. The poor outcome scores highlight the burden of the complication of glenoid loosening.^25^

Upon analyzing general data of reinterventions, instability and infection were determined to be the most common indications for reintervention and accounted for approximately 50% of all indications.^4^, ^5^, ^6^ However, according to our data, those complications needed revision to hemiarthroplasty to a much lesser degree (2 out of 12 patients).

To date, only one comparable study^16^ exists with the inclusion of 16 patients, of whom 14 were available at 6 weeks for clinical and radiological follow-up, 11 were available at 2 years, and 3 were available at 5 years. The patients reached similar results with an aCS of 25 ± 12 points, and almost half of the patients were dissatisfied. Interestingly, and not in accordance with our data, they could not detect a significant change in the pain level after revision to hemiarthroplasty with stable visual analog scale pain scores of 5/10 preoperatively and postoperatively. Our data showed improvement of at least a mean of 5 Constant-Murley pain score points (from 7 ± 3 to 12 ± 3 [0 worst, 15 pain-free]), with half of the patients reporting being completely pain-free at the final follow-up visit. Pain might be the main reason for revision surgery in such devastating cases where RTSA retention does not seem possible. We consider that result to be one of this study's most valuable pieces of information for patients and the treating physicians.

In our cohort, the postoperative function was very limited, with approximately 50° of flexion and abduction, which is in line with the data of Glanzmann et al.^16^ This result is significantly worse than an RTSA without reintervention surgery; most patients were still able to perform simple activities of daily living, such as eating, grooming, combing hair, or simple household tasks.^27^^,^^35^

Gamradt et al reported similar results in a case series of 6 patients with a follow-up duration of 26.5 months (minimum-maximum, 10-41 months) after revision to hemiarthroplasty.^13^ The ROM was comparable to that of the study by Glanzmann et al^16^ and our data, with an average elevation of 50°. They reported a generally low pain level (2.4/6) in their cohort. Glenoid bone grafting was performed in 4 of 6 patients with the option of conversion back to RTSA at a later stage. However, no patient elected to undergo this procedure.^13^ This is something we also observed in our patient cohort, as we identified 7 of 12 patients in whom glenoid bone grafting was performed in view of a secondary glenoid implantation. However, this was never executed, mainly after an informed decision-making process between the treating physician and the patient. They generally already underwent many reintervention surgeries and were, at least to a certain degree, satisfied with the achieved definitive treatment at this point. Kilian and Edwards^21^ proposed another approach termed “reverse hemiarthroplasty.” The authors performed glenoid bone grafting and implemented the RTSA glenoid component without implantation of the humeral stem in the first stage. At the time of glenoid component consolidation, the humeral stem was implanted in a second stage surgery. This approach might be useful in patients with planned stem exchange.

Some limitations have to be noted. (1) The study retrospectively investigated a single institution's cohort based on a prospective data collection. (2) The number of patients who underwent hemiarthroplasty revision was very small, including 21 patients, of whom only 12 had sufficient clinical follow-up of more than 2 years after revision surgery. The patients were older in many cases, and three patients in our cohort died before follow-up. Nevertheless, the cohort is the largest reported in the current literature. (3) Furthermore, the patients analyzed were heterogeneous, with different primary indications for RTSA and different secondary indications for revision to a hemiarthroplasty. More studies are necessary to analyze the outcomes according to the different indications and compare different salvage strategies. (4) Finally, different humeral head implants were used (ie, standard hemiarthroplasty heads versus bipolar big head hemiarthroplasty implants). The implants were chosen based on the surgeon's preference depending on the size of the dead space and bony- and soft-tissue deficiencies. Owing to the limited numbers, we could not perform a subgroup analysis to show the superiority of one implant over the other.

## Conclusion

Failed RTSA that is not feasible to retain is a burdensome complication with inferior results compared to patients with RTSA retaining revision surgery or patients without reintervention. In which cases, RTSA preserving surgery should be performed, and conversion to hemiarthroplasty is required and needs further investigation. According to this study, RTSA conversion to a hemiarthroplasty is a valid fallback-treatment option providing limited shoulder function but allowing the execution of some activities of daily living and, most importantly, reduces patients' pain levels to a certain degree.

## Disclaimers

*Funding:* No funding was disclosed by the authors.

*Conflicts of interest:* The authors, their immediate families, and any research foundation with which they are affiliated have not received any financial payments or other benefits from any commercial entity related to the subject of this article.
