# Supplementary material for: Hemiarthroplasty as a salvage treatment for failed reverse total shoulder arthroplasty
Source: JSES Int. 2021 Sep 4;5(6):1055–61. doi: 10.1016/j.jseint.2021.07.003 (PMC8568842; doi:10.1016/j.jseint.2021.07.003)
Supplement: Supplemental Table 1 [file mmc1.docx]

**Supplemental Table 1:** Displays pre- and postoperative outcome measures as mean ± standard deviation (range min to max) for the RTSA with conversion to hemiarthroplasty, revision RTSA with retaining components and the control group.

|  | | **Hemi** | **p** | **REV CTL** | **p** | **CTL** | **p** |
| --- | --- | --- | --- | --- | --- | --- | --- |
| **Number** | | 12 |  | 12 |  | 12 |  |
| **aCS** | |  |  |  |  |  |  |
| Preop RTSA | 30 +- 11 | 0.53 | 32 ± 9 | 0.00 | 35 ± 15 | 0.00 |  |
| Preop Revision | 30 +- 18 | 0.75 | 26 ± 13 | 0.03 |  |  |  |
| Latest FUP | 33 +- 10 |  | 55 ± 19 |  | 69 ± 12 |  |  |
| **rCS (%)** | |  |  |  |  |  |  |
| Preop RTSA | 39 +- 14 | 0.62 | 40 ± 12 | 0.00 | 45 ± 17 | 0.00 |  |
| Preop Revision | 39 +- 21 | 0.79 | 33 ± 16 | 0.02 |  |  |  |
| Latest FUP | 42 +- 14 |  | 67 ± 20 |  | 84 ± 13 |  |  |
| **SSV (%)** | |  |  |  |  |  |  |
| Preop RTSA | 28 +- 18 | 0.35 | 27 ± 15 | 0.00 | 37 ± 17 | 0.00 |  |
| Preop Revision | 37 +- 25 | 0.96 | 27 ± 22 | 0.04 |  |  |  |
| Latest FUP | 35 +- 19 |  | 64 ± 20 |  | 81 ± 15 |  |  |
| **CMS Pain** | |  |  |  |  |  |  |
| Preop RTSA | 6 +- 4 | 0.00 | 8 ± 4 | 0.01 | 5 ± 4 | 0.00 |  |
| Preop Revision | 7 +- 4 | 0.00 | 7 ± 4 | 0.01 |  |  |  |
| Latest FUP | 12 +- 3 |  | 13 ± 3 |  | 13 ± 3 |  |  |
| **Flexion (°)** | |  |  |  |  |  |  |
| Preop RTSA | 65 +- 27 | 0.09 | 69 ± 38 | 0.08 | 97 ± 31 | 0.02 |  |
| Preop Revision | 75 +- 36 | 0.34 | 54 ± 25 | 0.05 |  |  |  |
| Latest FUP | 55 +- 27 |  | 102 ± 34 |  | 128 ± 24 |  |  |
| **Abduction (°)** | |  |  |  |  |  |  |
| Preop RTSA | 63 +- 25 | 0.02 | 65 ± 26 | 0.02 | 78 ± 25 | 0.00 |  |
| Preop Revision | 70 +- 39 | 0.31 | 53 ± 23 | 0.04 |  |  |  |
| Latest FUP | 50 +- 23 |  | 109 ± 42 |  | 142 ± 24 |  |  |
| **ER (°)** | |  |  |  |  |  |  |
| Preop RTSA | 23 +- 26 | 0.16 | 27 ± 26 | 0.31 | 28 ± 25 | 0.93 |  |
| Preop Revision | 9 +- 30 | 0.82 | 21 ± 23 | 0.52 |  |  |  |
| Latest FUP | 13 +- 23 |  | 17 ± 17 |  | 28 ± 27 |  |  |
| **IR** | |  |  |  |  |  |  |
| Preop RTSA | 5 +- 3 | 0.73 | 4 ± 2 | 0.78 | 5 ± 3 | 0.07 |  |
| Preop Revision | 3 +- 3 | 0.17 | 3 ± 2 | 0.55 |  |  |  |
| Latest FUP | 5 +- 3 |  | 4 ± 2 |  | 7 ± 2 |  |  |
| **Force mean (kg)** | |  |  |  |  |  |  |
| Preop RTSA | 0 +- 1 | 0.19 | 1 ± 2 | 0.13 | 1 ± 1 | 0.02 |  |
| Preop Revision | 1 +- 1 | 0.06 | 0 ± 1 | 0.07 |  |  |  |
| Latest FUP | 0 +- 0 |  | 2 ± 2 |  | 3 ± 2 |  |  |
| **Follow-up (m)** | |  |  |  |  |  |  |
| Post Revision | 46 ± 26 |  | 61 ± 29 |  |  |  |  |
| Post RTSA | 66 ± 30 |  | 72 ± 25 |  | 80 ± 33 |  |  |

P-Values show the comparison to the latest follow-up using the Wilcoxon ranksum test.

Abbreviations: CMS – Constant-Murley Score; CTL – Control; ER – External rotation; Hemi – Hemiarthroplasty; IR – Internal rotation; kg – kilogram; postop – postoperative; preop – preoperative; RTSA – Reverse Total Shoulder Arthroplasty; SSV – Subjective Shoulder Value.
